# De-identifying Spanish medical texts - named entity recognition applied to radiology reports

**DOI:** 10.1186/s13326-021-00236-2

**Published:** 2021-03-29

**Authors:** Irene Pérez-Díez, Raúl Pérez-Moraga, Adolfo López-Cerdán, Jose-Maria Salinas-Serrano, María de la Iglesia-Vayá

**Affiliations:** 1 FISABIO-CIPF Joint Research Unit in Biomedical Imaging. Fundació per al Foment de la Investigació Sanitària i Biomèdica (FISABIO), Av. de Catalunya 21, València, 46020 Spain; 2 Bioinformatics and Biostatistics Unit. Centro de Investigación Príncipe Felipe (CIPF), Carrer d’Eduardo Primo Yúfera 3, València, 46012 Spain; 3 ESI International Chair@CEU-UCH, Departamento de Matemáticas, Física y Ciencias Tecnológicas, Universidad Cardenal Herrera-CEU, CEU Universities, Calle San Bartolomé 55, Alfafara del Patriarca, 46115 Spain; 4 Health Informatics Department, Hospital San Juan de Alicante, Sant Joan d’Alacant, 03550 Spain; 5 Regional ministry of Universal Health and Public Health in Valencia, Carrer de Misser Mascó 31, València, 46010 Spain; 6 CIBERSAM, ISCIII, Av. Blasco Ibáñez 15, València, 46010 Spain

**Keywords:** Natural language processing, Named entity recognition, Radiology reports, Medical texts, Spanish

## Abstract

**Background:**

Medical texts such as radiology reports or electronic health records are a powerful source of data for researchers. Anonymization methods must be developed to de-identify documents containing personal information from both patients and medical staff. Although currently there are several anonymization strategies for the English language, they are also language-dependent. Here, we introduce a named entity recognition strategy for Spanish medical texts, translatable to other languages.

**Results:**

We tested 4 neural networks on our radiology reports dataset, achieving a recall of 97.18% of the identifying entities. Alongside, we developed a randomization algorithm to substitute the detected entities with new ones from the same category, making it virtually impossible to differentiate real data from synthetic data. The three best architectures were tested with the MEDDOCAN challenge dataset of electronic health records as an external test, achieving a recall of 69.18%.

**Conclusions:**

The strategy proposed, combining named entity recognition tasks with randomization of entities, is suitable for Spanish radiology reports. It does not require a big training corpus, thus it could be easily extended to other languages and medical texts, such as electronic health records.

## Background

Medical imaging is widely used in clinical practice for the diagnosis and treatment of several diseases, such as Alzheimer, cancer or pneumothorax. Data from radiology reports, electronic health records and other medical texts such as clinical trial protocols are being used for research purposes [1, 2]. Health care institutions, researchers and patients can greatly benefit from these datasets. However, these records and reports contain patient notes known as personal data that can challenge patient confidentiality and privacy, as provided for in the European Regulation on the protection of personal data [3]. All words that could identify a patient must be removed or de-identified before data analysts start their research or even more before the dataset is published.

From a legal point of view, Regulation (EU) 2016/67 on the protection of natural persons and with regard to the processing of personal data and on the free movement of such data [3] provides the regulatory framework in the European Union. Although its application is mandatory to all its member states, its concrete implementation varies depending on each of them. In Spain, the Organic Law 3/2018 [4] establishes the legal framework for data protection in biomedical research. Reuse of personal data for medical research needs to be approved by an ethics committee, and data must be at least pseudonymized before the researchers get access to it.

Legal issues regarding data privacy are not the only source of concern. Direct consequences for patients are also a very important factor to be carefully considered. It is crucial to protect the private health details of a patient from any third party’s access, and avoid exposing identifiable personal data such as identifier numbers or addresses. De-identification is therefore essential to ensure patient privacy and comply with legal requirements.

From a data management point of view, the de-identification methodology needs to be precise and recallable. Precision is needed to minimize the data loss of the de-identification process and to preserve the semantic meaning of the radiology report; recall allows getting the best de-identification possible and avoid leaving any identifiable information in the text [5].

Even though several de-identification or anonymization methodologies have been proposed in English, legislation differs on a national level worldwide and language-specific problems can arise, hence a different method for each language must be developed. These difficulties extend to any Natural Language Processing (NLP) implementation. In the biomedical field, NLP has been applied successfully in English, including for de-identification purposes [6], but many of these strategies rely on language-specific resources and are not extensible to other languages [7]. Apart from the English language, this problem has been assessed in French, where different strategies from machine learning to the use of dictionaries and lists have been proposed, along with protocols for corpus development [8, 9]. In other languages such as German, Swedish, Dutch or Chinese some strategies and methodologies have also been proposed [5, 10–13], but there have been so far rather limited attempts in automatic de-identification for Spanish medical texts [14–16], such as the MEDDOCAN task [16]. For the sake of giving an insight on the different approaches proposed by these authors, the datasets used and the performance of each work, we have summarized this information in Table 1.

**Table 1:** State of the art summary for de-identification studies in non-English languages

Study	Methodology	Recall	F1-score	Corpus size	Identifying tokens
Dalianis et al. [5]	CRF	0.715	0.810	100 clinical records, train set	6170
				4-fold cross-validation	
Menger et al. [12]	Regular expression rules	0.916	0.862	2000 medical texts, development	542, test set
	and tree-based hashing			400 medical texts, test set	
Jian et al. [13]	Rule-based and CRF	0.851	0.848	201 sentences, train set	1259, train set
				1000 clinical records, test set	
Lange et al. [28]	BiLSTM with CRF	0.974	0.974	500 clinical records, train set	11333, train set
				250 clinical records, development	5801, development
				250 clinical records, test set	5661, test set
Jiang et al. [29]	BERT and flair system	0.968	0.962	500 clinical records, train set	11333, train set
				250 clinical records, development	5801, development
				250 clinical records, test set	5661, test set
Pérez et al. [30]	spaCy	0.953	0.960	500 clinical records, train set	11333, train set
				250 clinical records, development	5801, development
				250 clinical records, test set	5661, test set

The table describes the methodology used by the authors, the performance of the approach and the corpus size in number of documents and number of identifying tokens. From MEDDOCAN, the top 3 best-performing models were included

Most of the works around text de-identification are based on pattern matching or machine learning, or even a combination of both. Whereas pattern matching does not account for the context of a word and is unaware of typographical errors, machine learning techniques require a large corpus of annotated text [17]. Since our radiology reports were mostly free text with sensitive data outside headers, we opted for annotating our own corpus and developing a Named Entity Recognition (NER) based de-identification method.

NER is a sequence tagging task comprised inside the field of NLP, which focuses on assigning different tokens or words into specific predefined classes, such as persons, dates or organizations. NLP tasks are usually based on recurrent neural networks (RNNs), and NER approaches tend to employ long short-term memory units (LSTM) [18] combined with conditional random fields (CRF) [19, 20]. LSTMs are variants of RNNs that can cope with long distance dependencies in the text, and for many applications it is beneficial to access to left and right context in the sentence through bi-directional LSTMs [20, 21]. Moreover, the reference model for several state-of-the-art NER implementations in English language is the bidirectional LSTM (BiLSTM)-CRF model by Lample et al. [22–24]. Some implementations combine LSTM units with convolutional layers [24, 25], and other architectures such as Bidirectional Encoder Representations for Transformers (BERT) [26] have been proposed for several NLP tasks, including NER.

Although some contests and projects have been organized to exploit the content of unstructured clinical records in Spanish language using NLP tools, they are not focused on de-identification. For example, Cantemist (Cancer Text Mining SharedTask) is a project held to gather a community effort to create tools and models to perform text mining using NLP in oncological records [27]. The best performing models in this contest were based on BiLSTM with CRF. Nevertheless, regarding the de-identification of clinical text for secondary use, in 2019 the MEDDOCAN (Medical Document Anonymization) task was organized. The most successful models in this task employ deep learning-based methodologies to perform a NER detection task, for instance, the winner model presented by Lange et al. [28] used a network based on BiLSTM-CRF and achieved a recall and F1 score of 0.974. The second-best model for the de-identification task was designed by Jiang et al. [29] with a model based on BERT and Flair embeddings, and achieved a recall of 0.962 and a F1 score of 0.968. The third proposed model used a spaCy NER model achieving a recall of 0.953 and F1 score of 0.960 [30].

Having in mind that the best NER approaches in Spanish language and in the general literature are based on RNNs with LSTM units and CRF, we decided to focus our work on these architectures. Nevertheless, automatic de-identification approaches do not achieve a perfect recall score, meaning that sensitive information could be leaked. To address this issue, we have proposed and developed a methodology to combine both NER and the replacement of the named entities recognized with synthetic data.

## Methods

**Fig. 1:**
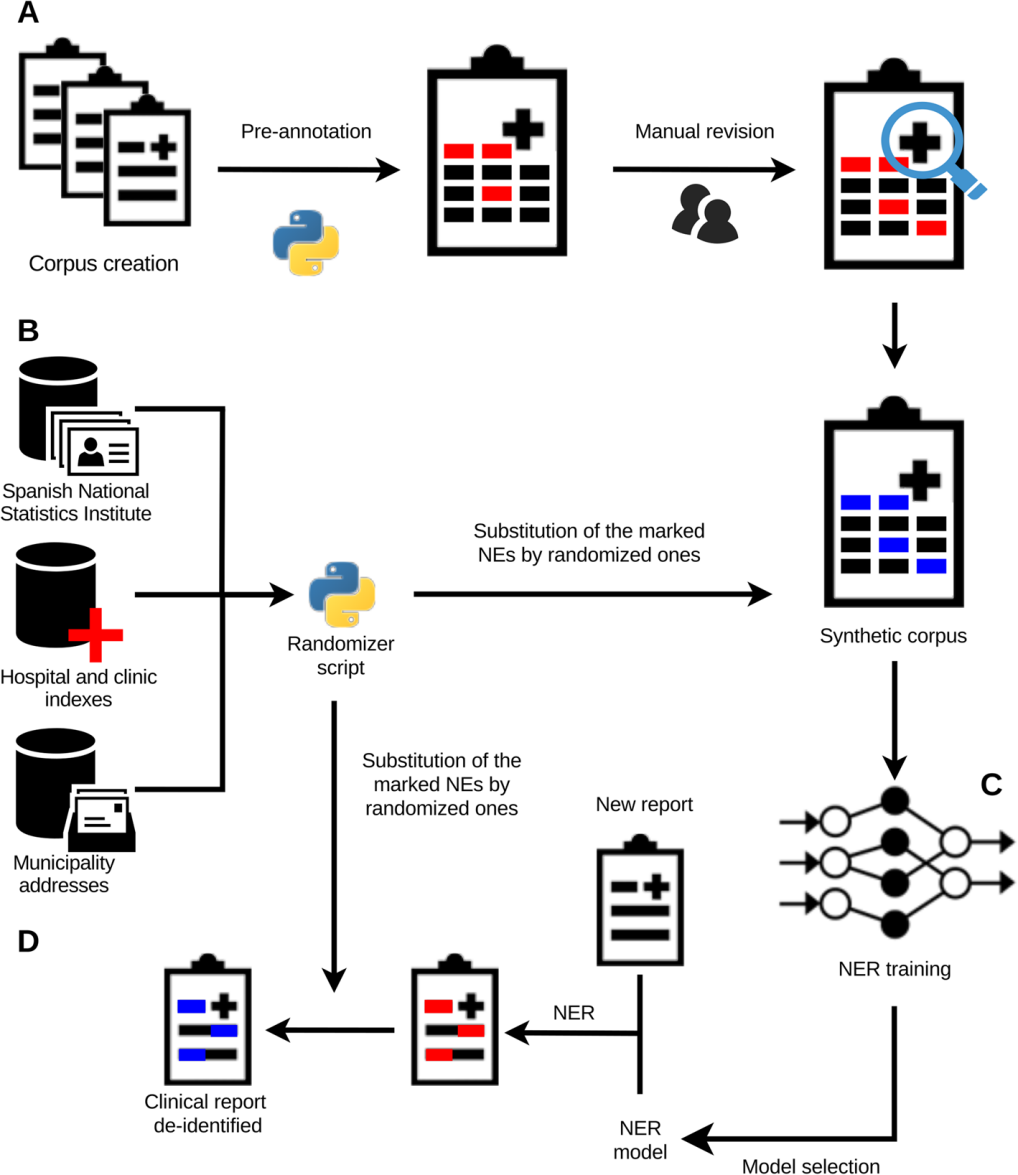
Summary of the proposed de-identification approach. **a** Corpus creation, annotation and manual revision, further detailed in Fig. 2. **b** Selection of databases to develop a randomizer script. The script is used to create the synthetic corpus. **c** Training and testing of different neural networks to select the best performing model. **d** When a new report needs to be de-identified, the selected model labels the words that belong to one of the defined named entities. Finally, the randomizer script creates a de-identified report with synthetic information

The proposed methodology is based on a combination of NER and the substitution of the detected sensitive words with others randomly sampled from databases. The approach started with the definition of the named entities that contain sensitive information and the annotation of the corpus (Fig. 1a). Then, a randomizer script was created based on publicly available databases to create a synthetic corpus by substituting the manually annotated words by new ones extracted from the databases (Fig. 1b). This corpus was then fed to different NER neural networks to assess their performance and select the most suitable model for the desired application (Fig. 1c). Lastly, when a new radiology record needs to be de-identified, the trained model detects the named entities and the randomizer script substitutes them with random words of the same category (Fig. 1d).

### Named entities

Given that there is no specific guidance in the Spanish legal system on what information has to be removed to de-identify medical texts, we decided to assess the presence in our corpus of the Protected Health Information (PHI) categories defined by the Health Insurance Portability and Accountability Act (HIPAA) in the United States of America [31]. After manual inspection of the data and considering the scope of this work, we performed a sub-selection of PHI categories and finally grouped them in 6 Named Entities (NEs) as shown in Table 2. Some NEs included other information that should be protected to preserve the privacy of patients or doctors but was not included in PHI categories, such as digital signatures or healthcare centres. The named entities selected were:

- NAME (name): This NE includes names and surnames of any person mentioned in the radiology record, typically patients or medical staff.

**Table 2:** Named entities selected for this task and their associated Protected Health Information categories

NEs	Description	PHIs
CAB	Section headers	-
NAME	Names and surnames (patient and others)	Names
DIR	Full addresses, including streets, numbers and zip codes	Geographic data
LOC	Cities, inside and outside addresses	Geographic data
NUM	Numbers or alphanumeric strings that might identify someone, including digital signatures, patient numbers, medical numbers, medical license numbers and others	medical record numbers, social security numbers, account numbers, any unique identifying number or code
FECHA	Dates	Dates
INST	Hospitals, healthcare centres or other institutions that might point to someone’s location	-
- DIR (address): Includes geographic data in form of full addresses, including streets and zip codes.
- LOC (locations): Considers geographic data referring only to cities, villages and other populated areas. This is differentiated from the DIR named entity due to the possibility of a city to be mentioned out of the context of a full address, for example, next to a data as in “14 de Abril, Valencia”.
- NUM (numbers): Includes any number or alphanumeric string that might identify a person, such as patient record identification numbers, medical license numbers, digital signatures, fiscal identification numbers and others.
- FECHA (dates): Any date available in the report, either numeric or written.
- INST (institutions): Any healthcare facility or institution mentioned in the radiology record that could be used to narrow the location of a patient or medical staff.

Header sections (CAB) were included as a seventh NE to ensure that they were not removed from the final text. These headers are necessary for further analysis, being key to extract the most relevant information of a radiology report.

### Corpus construction

**Fig. 2:**
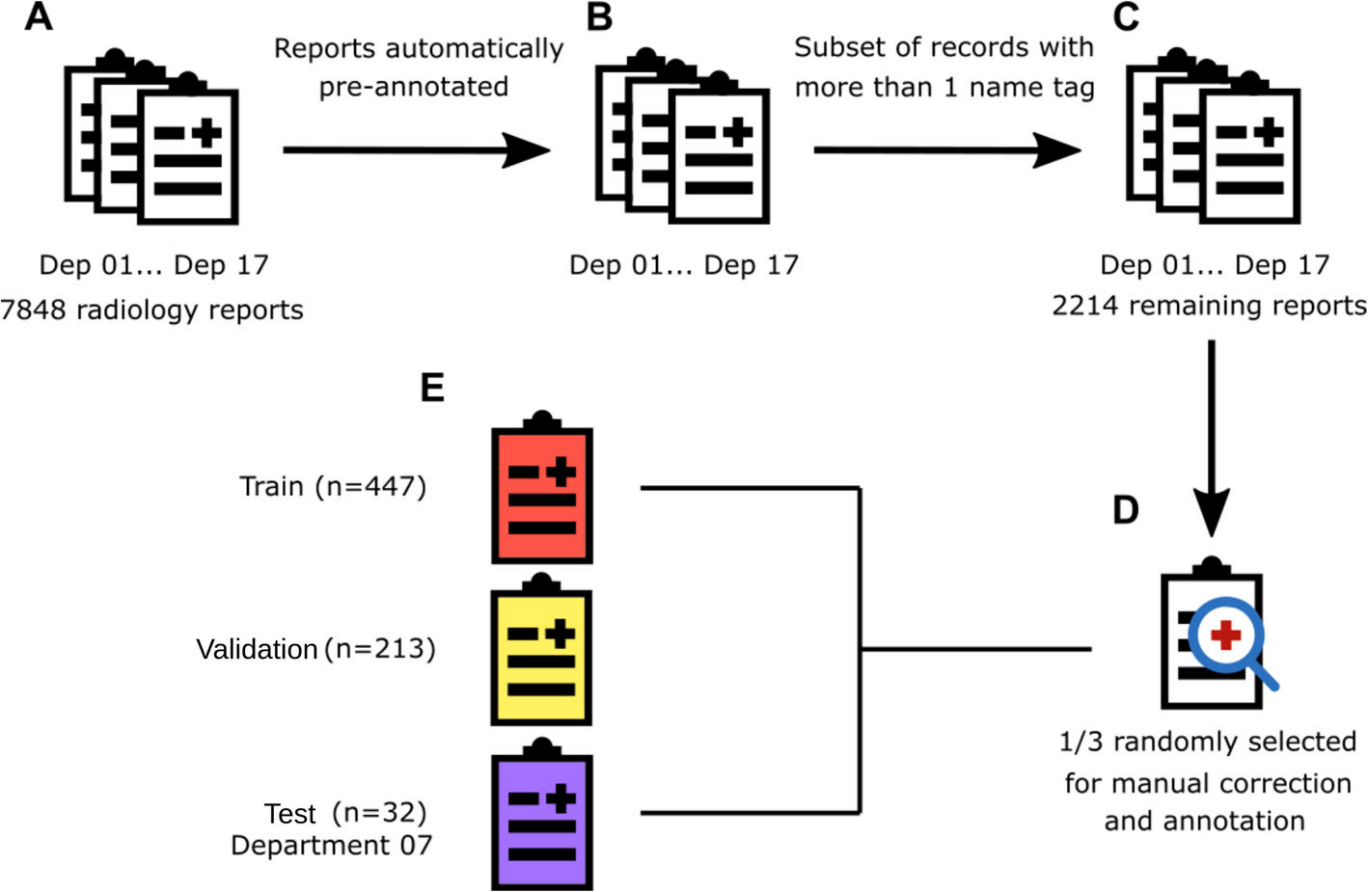
Data curation process and corpus preparation workflow. **a** 7848 radiology reports in total were retrieved from BIMCV database. **b** We used a custom Python script to automatically annotate the names, surnames and hospital names from radiology reports. **c** A subset of records was made meeting the condition that more than one ‘name’ tag was present, remaining 2214 reports. **d** Another subsetting was performed to randomly select one-third of reports to be manually annotated and corrected by three annotators. After the manual revision, 692 reports remain. **e** Ground Truth dataset was divided into 3 subsets: the training set included 447 reports, validation 213, and test 32 reports from healthcare department number 7

The de-identification corpus consists of brain imaging radiology reports randomly extracted from the Medical Imaging Databank of the Valencian Region (BIMCV) database [32, 33], distributed among 17 health departments of the Valencian Region (Fig. 2). A total of 7848 records were initially retrieved and automatically pre-annotated using the Spanish National Statistics Institute name and surname database [34], which includes those names with a frequency higher or equal to 20 in Spain, and a list of the hospital names in the Valencian Region. To ensure the presence of personal information in our corpus, a subset of reports with at least two “NAME” tags was extracted. This filter left out of the selected reports those including words like “cabeza”, included in the text as an anatomical part although it can be also a surname, but containing no sensitive information. One-third of those reports were randomly selected to be manually corrected and annotated, with a final corpus of 692 records. The annotations were manually reviewed by three annotators, including finally all the NE tags.

**Fig. 3:**
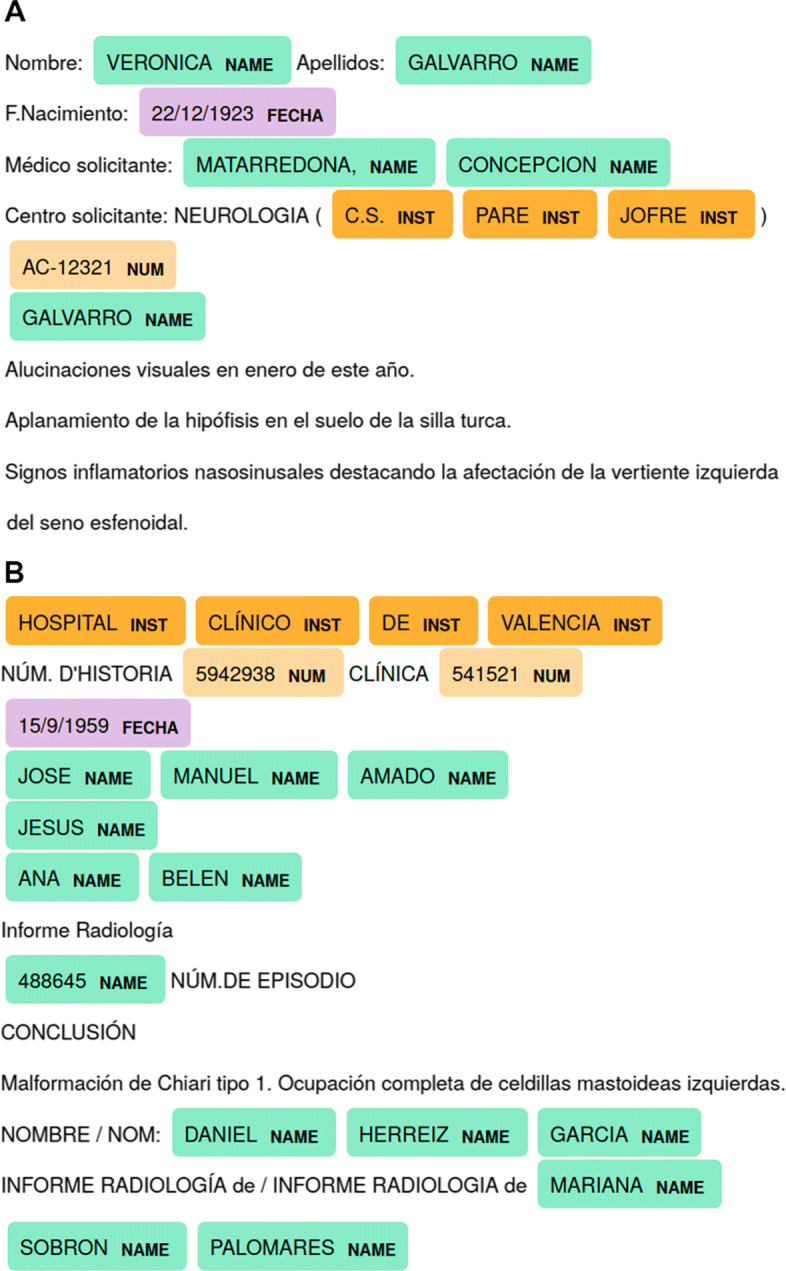
Partial examples of radiology reports from validation and test. Validation set (**a**) has metadata headers clearly defined. In turn, test set (**b**) has metadata headers in Valencian language and metadata information detached from these headers by a line break. Both structures include identifiable information in new lines without metadata headers. Any name, surname, address, identification number or date presented in the figure are fictitious
- Validation set, including 213 randomly selected records from all the departments except 7th department.
- Test set, including 32 randomly selected records from the 7th department.

Radiology reports were not pre-processed so that they remain unchanged after the de-identification, apart from the identifying information. Although our radiology reports were mostly free-text sections preceded by headers, the 7th health department lacked headers and had an increased number of entities entirely out of context: this is, a name or a surname with no more text in an independent line, as shown in Fig. 3. With this in mind, we divided our dataset into three sets:

- Training set, including 447 randomly selected records from all the departments, including 65 reports from the 7th health department.

**Fig. 4:**
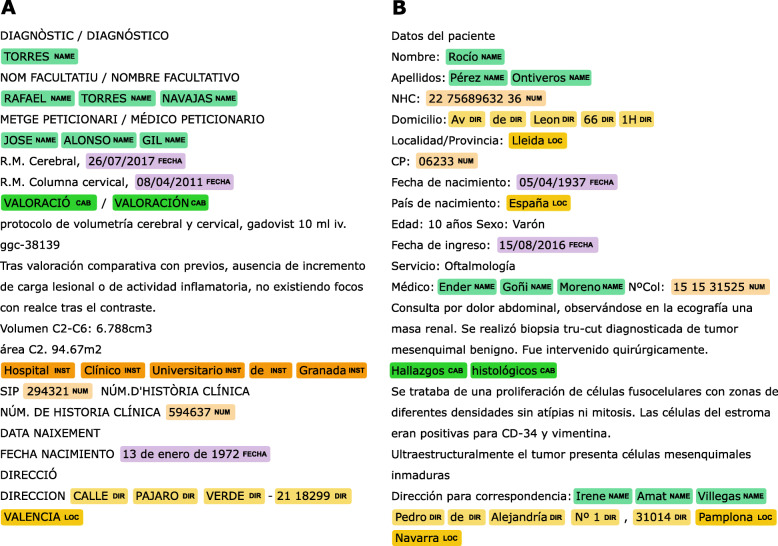
Structure differences between the radiology records used for training/testing and the clinical records from MEDDOCAN. **a** Radiology record from the Valencian Region, where names, surnames and other sensitive information from patients and medical staff are not always in the same line that the metadata information. **b** Clinical record from MEDDOCAN, where sensitive data is preceded by their correspondent metadata descriptors. Any name, surname, address, identification number or date present in the figure are fictitious

To assess the performance of our final model with external data, we decided to incorporate 100 randomly selected clinical records from the MEDDOCAN task [16]. These records have a different structure (Fig. 4) and are not related to radiology.

Whereas both training and validation sets present a similar distribution of NEs (Table 3), the test set shows an increase of addresses, locations and institutions. Having a separate test for department 7 allows us to check the performance of our method with highly unstructured data, with a distribution of NEs different from the training. As shown in Table 3, addresses and locations are the NEs with the lowest sample size.

**Table 3:** Number and percentage of annotations per corpus subset: Training, validation and test

	Training (words / %)	Validation (words / %)	Test (words / %)
CAB	1987 / 21.37%	993 / 20.87%	120 / 9.4%
NAME	3286 / 35.34%	1591 / 33.45%	386 / 30.25%
DIR	128 / 1.38%	106 / 2.23%	72 / 5.64%
LOC	79 / 0.85%	46 / 0.97%	26 / 2.04%
NUM	1159 / 12.47%	585 / 12.29%	143 / 11.21%
FECHA	1655 / 17.79%	897 / 18.86%	300 / 23.51%
INST	1004 / 10.80%	539 / 11.33%	229 / 17.95%

### NE randomization

We developed a methodology to randomize the PHIs found in a text, and applied it to the manually labelled dataset, obtaining a synthetic corpus. This methodology applies a set of rules depending on the NE associated with each tagged word. It is based on the substitution of tagged entities with new words randomly extracted from different databases available online:

- Spanish National Statistics Institute name and surname database [34], weighted by frequency. This database includes foreign names and surnames, such as Xiaojing, Steven, Abdul or Harrison.
- Spanish National Statistics Institute municipal register database [35], weighted by population in 2019.
- National Hospital Index [36].
- National Outpatients Clinic Index [37].
- Municipality addresses [38].

With the aim of avoiding the leakage of sensitive personal data, this methodology also checks that the randomly chosen word or number is not the same as the original one.

### Networks

A variety of neural networks were tested and evaluated, all of them designed for NER tasks. Three network architectures were based on Bidirectional Long Short-Term Memory (BiLSTM) layers, obtained from Guillaume Genthial’s GitHub repository [39]:

- LSTM-CRF: GloVe vectors, BiLSTM and Conditional Random Fields (CRF) based on the work of Huang *et al* [20].
- LSTM-LSTM-CRF: GloVe vectors, character embeddings, BiLSTM for character embeddings, BiLSTM and CRF, based on the work of Lample *et al* [22].
- Conv-LSTM-CRF: GloVe vectors, character embeddings with 1D convolution and max pooling, BiLSTM and CRF, based on the work of Ma and Hovy [40].

These networks were trained with and without Exponential Moving Average (EMA) of the weights. We also trained a spaCy [24] NER model, based partly on the work of Lample *et al* [22] with Bloom embeddings along with Convolutional Neural Networks (CNNs) with an attention mechanism.

### Evaluation metrics

To assess the performance of the different models trained we computed precision, recall and F1-score metrics. These metrics can be defined as:

$$precision = \frac{TP}{TP + FP} $$

$$recall = \frac{TP}{TP + FN} $$

$$F1 score = \frac{2 \cdot precision \cdot recall}{precision + recall} $$

being TP the number of true positives, FP the number of false positives, and FN the number of false negatives.

To compute the amount of de-identification achieved by the model, we did not only apply these metrics to each NE, but to the set of words that should have been labelled as an identifying NE. With this approach, we obtained quantitative indicators of global de-identification.

## Results

First, models for each neural network were trained and then evaluated. Table 4 shows the mean global results of the different networks, given three replicates for each one.

**Table 4:** Evaluation metrics for each of the different neural networks tested

	Training set			Validation set			Test set		
Model	Precision	Recall	F1	Precision	Recall	F1	Precision	Recall	F1
LSTM-CRF	90.39	81.93	85.95	87.09	77.11	81.79	81.35	61.37	69.96
LSTM-CRF with EMA	91.19	84.15	87.53	87.05	78.49	82.55	71.48	59.65	64.96
LSTM-LSTM-CRF	99.20	98.79	98.99	**98.13**	97.18	97.66	93.01	90.94	91.96
LSTM-LSTM-CRF with EMA	99.06	98.96	99.01	98.00	97.34	97.67	94.20	**91.10**	**92.63**
Conv-LSTM-CRF	99.31	99.05	99.18	98.11	97.29	97.70	**94.49**	90.43	92.41
Conv-LSTM-CRF with EMA	99.17	99.05	99.11	98.08	**97.36**	**97.72**	93.72	90.64	92.15
Spacy	**99.87**	**99.28**	**99.58**	98.06	96.10	97.07	93.23	89.39	91.31

Bold font highlights the best metric in each data subset

The recall is one of the most relevant evaluation metrics in any de-identification process [5], to avoid the leakage of sensitive information. Taking this into account, LSTM-LSTM-CRF with EMA shows the highest recall in test, and Conv-LSTM-CRF with EMA in validation. Although these are the two best-performing networks in both sets, we decided to include also spaCy for further analysis and leave outside the worst-performing architecture: LSTM-CRF.

The performance stats of each NE for LSTM-LSTM-CRF with EMA, Conv-LSTM-CRF with EMA and spaCy are displayed in Tables 5, 6 and 7. Whereas in training set spaCy outperforms the other networks in every NE except for CAB, in validation and test sets the results are more contested. Evaluating F1-score in validation, LSTM-LSTM-CRF classifies better dates, locations, names and numbers, while spaCy stands out with institutions. On the other hand, Conv-LSTM-CRF performs better with addresses and shows higher recall in names than LSTM-LSTM-CRF. When analysing the results for the test set, the spaCy model shows better metrics in dates and better recall in institutions whereas LSTM-LSTM-CRF has a higher F1-score in institutions, locations and names. Conv-LSTM-CRF again performs better with addresses, but also with numbers and shows the highest recall in locations and names. When applying the models to MEDDOCAN dataset there’s a decay of the performance, although spaCy has higher recall rates in addresses, dates, institutions and name, whilst Conv-LSTM-CRF outperforms in locations and numbers.

**Table 5:** Evaluation metrics obtained with LSTM-LSTM-CRF with EMA model for each named entity

	Training set			Validation set			Test set			MEDDOCAN		
	Precision	Recall	F1	Precision	Recall	F1	Precision	Recall	F1	Precision	Recall	F1
CAB	98.29	97.94	98.11	96.03	94.92	95.47	83.69	75.76	79.53	13.33	33.33	19.05
DIR	100	100	100	93.49	95.00	94.22	90.91	90.91	90.91	0.00	0.00	0.00
FECHA	99.74	99.64	99.69	98.93	99.20	99.07	96.65	94.83	95.74	74.95	86.34	80.20
INST	98.96	98.96	98.96	95.73	95.72	95.73	96.08	96.08	96.08	11.11	0.67	1.26
LOC	100	89.45	94.42	94.35	87.88	90.99	92.58	55.55	69.41	0.00	0.00	0.00
NAME	98.99	99.15	99.07	98.97	98.24	98.60	94.78	95.13	94.95	61.62	77.39	68.59
NUM	99.39	99.91	99.65	99.34	98.69	99.01	96.65	97.66	97.15	56.93	68.28	62.05
	99.05	98.96	99.01	98.00	97.34	97.67	94.20	91.10	92.62	62.35	56.11	59.07

**Table 6:** Evaluation metrics obtained with Conv-LSTM-CRF with EMA model for each named entity

	Training set			Validation set			Test set			MEDDOCAN		
	Precision	Recall	F1	Precision	Recall	F1	Precision	Recall	F1	Precision	Recall	F1
CAB	98.57	97.99	98.28	96.82	94.97	95.89	91.45	76.89	83.54	0.78	16.67	1.48
DIR	100	100	100	98.33	95.00	96.63	93.94	93.94	93.94	10.71	1.18	2.11
FECHA	99.71	99.78	99.74	98.79	99.13	98.96	95.77	93.21	94.47	82.28	86.21	84.18
INST	98.96	98.96	98.96	96.35	95.94	96.14	92.91	94.12	93.51	0.00	0.00	0.00
LOC	100	91.56	95.59	92.89	87.88	90.29	89.44	55.56	68.50	20.83	0.58	1.12
NAME	99.17	99.28	99.23	98.69	98.28	98.49	92.31	96.26	94.23	70.17	77.39	73.56
NUM	99.35	99.88	99.62	98.98	98.63	98.80	95.59	95.57	95.58	64.53	78.29	70.69
	99.17	99.06	99.11	98.08	97.36	97.72	93.72	90.64	92.16	67.07	58.90	62.71

**Table 7:** Evaluation metrics obtained with spaCy model for each named entity

	Training set			Validation set			Test set			MEDDOCAN		
	Precision	Recall	F1	Precision	Recall	F1	Precision	Recall	F1	Precision	Recall	F1
CAB	99.43	96.54	97.96	98.28	93.98	96.08	92.54	74.49	82.52	4.76	33.33	8.33
DIR	100	100	100	94.28	63.96	76.01	87.79	74.77	61.46	43.15	4.47	8.01
FECHA	100	100	100	98.54	99.04	98.78	98.20	97.53	97.86	51.39	89.41	65.13
INST	99.97	99.96	99.98	98.19	97.24	97.71	93.50	98.00	95.69	45.72	12.28	19.27
LOC	100	100	100	76.64	54.66	63.80	61.04	26.85	36.79	7.19	0.32	0.59
NAME	100	99.99	99.99	98.34	98.28	98.31	88.78	94.29	93.19	75.62	83.91	79.23
NUM	100	100	100	97.81	95.65	96.72	95.11	87.56	91.18	68.50	60.32	63.99
	99.87	99.28	99.58	98.06	96.10	97.08	93.23	89.39	91.31	65.63	55.37	59.98

**Fig. 5:**
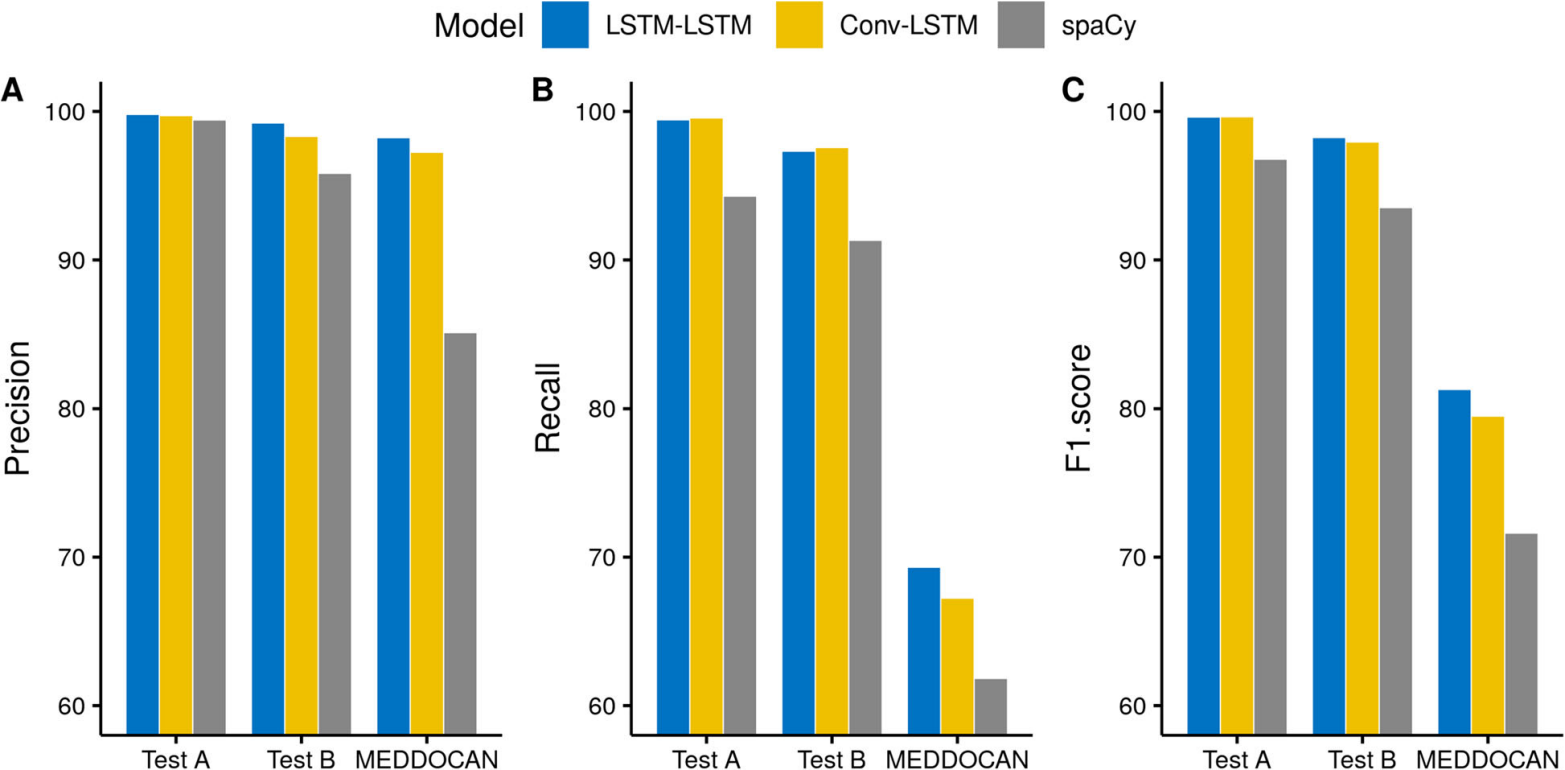
Global de-identification metrics for the three best performing architectures. Precision (**a**), recall (**b**) and F1-score (**c**) for the three best performing architectures, LSTM-LSTM-CRF with EMA (blue), Conv-LSTM-CRF with EMA (yellow) and spaCy (grey) by data subset

Given that our aim was not to correctly classify NE, but to completely remove sensitive information from the text, global de-identification metrics were computed (Table 8). Conv-LSTM-CRF with EMA shows better recall in validation and test sets (Fig. 5), whilst LSTM-LSTM-CRF has higher F1-score on test. On MEDDOCAN data, the model that better maintains recall and F1-score is LSTM-LSTM-CRF (Fig. 5, Table 8). To assess the performance of our models with external data, we wanted to apply the models generated at MEDDOCAN to our data. Only one of the participants made their models available [30], being one of the implemented networks spaCy. Their spaCy model achieved a precision of 87.89% and 80.31%, a recall of 42.66% and 26.54%, and an F1-score of 57.44% and 39.89% in our validation and our test, respectively (Table 8).

**Table 8:** Global de-identification metrics for LSTM-LSTM-CRF, Conv-LSTM-CRF, spaCy and the model retrieved from MEDDOCAN

	Validation set			Test set			MEDDOCAN		
	Precision	Recall	F1	Precision	Recall	F1	Precision	Recall	F1
LSTM-LSTM with EMA	99.66	99.29	99.48	99.08	97.18	98.10	98.09	69.18	81.13
Conv-LSTM-CRF with EMA	99.58	99.42	99.50	98.18	97.43	97.80	97.11	67.10	79.36
spaCy	99.28	94.15	96.64	95.69	91.18	93.38	84.96	61.69	71.48
MEDDOCAN model	87.89	42.66	57.44	80.31	26.54	38.89	96.70*	95.30*	96.60*

^(*)^Results extracted from the original publication [30]

## Discussion

This work has defined and evaluated a methodology based on NER to de-identify radiology reports in Spanish language. In comparison with traditional approaches based on regular expressions, NLP and neural networks do not underperform due to human misspellings or the absence of a clear and repeated structure. Neural networks are also context-dependent, and words like Cabeza (head), a common surname in Spanish that also refers to an anatomical part, will be detected as a “NAME” entity when used as a surname but left unchanged when used as a medical word, avoiding the loss of meaningful information.

The main drawback of this methodology is the requi- rement of a learning corpus of de-identified reports, which is not necessary for regular expression-based strategies. Although the curation of a corpus is a tedious and methodical task, there is no need for a big dataset: with a training set of 447 texts, we achieved a suitable performance.

Neural networks should be trained with a corpus diverse in structure to avoid overfitting. Machine learning models tend to learn the structure or format of the text, finding the position of words containing sensitive data when performing de-identification. If a model was trained with a corpus with a determined structure, it will only be able to de-identify similarly-formatted texts. By comparing our spaCy model with the spaCy model retrieved from MEDDOCAN [30], we show the high impact that text structure has in the outcome. The MEDDOCAN training set was similar in size to ours (500 and 447 texts with a median of 20 and 22 lines per text, respectively), but their text structure was highly defined and invariant (texts from both datasets are compared at Fig. 4). With a training set diverse in its structure we can obtain higher recall and precision in external data, generating a de-identification model better prepared to deal with new data. Figure 3 illustrates the structure and format diversity of radiological reports between health departments included in our dataset.

Considering that the recall metric assesses the capability to avoid the leakage of sensitive information of a model, we propose LSTM-LSTM-CRF with EMA as the best neural network to address a de-identification task based on NER. This neural network showed higher F1-score in the test and MEDDOCAN, and its recall in validation and test sets are comparable to those obtained with Conv-LSTM-CRF with EMA. Furthermore, its recall on MEDDOCAN outperforms the one obtained by other networks. Thus, we expect LSTM-LSTM-CRF with EMA to behave optimally when presenting new data to it. Although its recall is 99.29 and 97.18 for validation and test sets respectively, it is not perfect. If we compare the results obtained by our models with those presented in MEDDOCAN, our LSTM-LSTM-CRF trained model outperforms the winner of MEDDOCAN contest at F1-score, 98.1 vs 97.4, but not at recall level, 97.1 vs 97.4, respectively. Thus, our presented models are close in terms of performance with those models presented on MEDDOCAN.

**Fig. 6:**
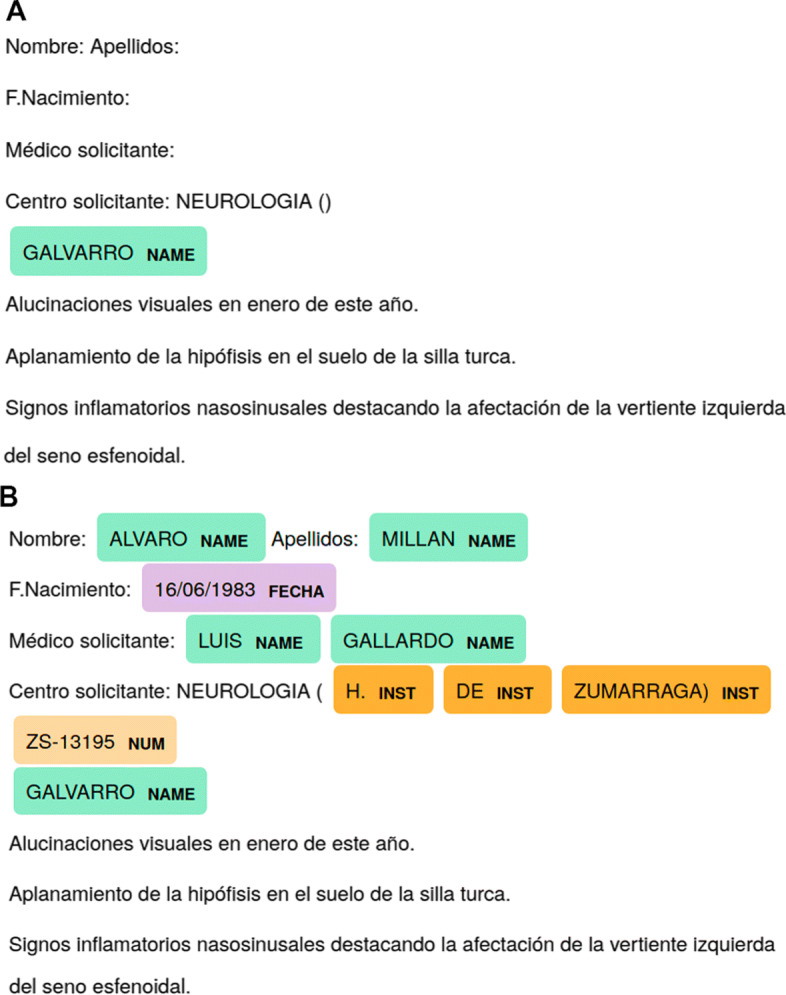
Anonymization strategies. When applying word elimination (**a**) errors are easily detectable whereas with synthetic substitution (**b**) any mistake is hidden with randomized synthetic information. Any name, surname, address, identification number or date presented in the figure are fictitious

When new radiology reports from the Valencian Region are included in BIMCV database, 97.18% of recall in test set means that almost 3% of identifying words will remain in the text. It might not be enough to re-identify the patient: could be left only a surname, a city name, or a part of an address. In fact, the de-identification methodology proposed in this work was applied to the COVID-19 image dataset described by de la Iglesia Vayá et al. [41], that needed to be reused for research due to the medical emergency situation in 2020. The radiology records in this dataset were revised by radiologists, finding in 28 out of 11558 (0.24%) reports enough sensitive information to identify patients or medical staff. This included names, patient record identification numbers, birthdates or healthcare centre names. To ensure that the identity of a patient is not recoverable, a final check of the texts by an authorized person remains necessary. Nevertheless, we propose a randomization strategy to change the identified NEs for synthetic ones of the same category. This strategy masks the identifying words left by the neural network with synthetic information, making it more difficult to discern between real and synthetic identifying words than by simply erasing words (Fig. 6). Further efforts need to be done to validate whether this strategy makes original information irretrievable or not.

## Conclusions

Medical texts hold great potential for research, but legal and privacy concerns arise with its use, even more, when institutions external to the hospital are involved. Real-world medical texts tend to be semi-structured with free text that includes sensitive information, thus classical de-identification approaches based on regular expressions are not good enough. We propose a robust and flexible framework based on NER for Spanish medical texts, tested on radiology reports from the Valencian Region. This framework is generic and relatively simple and can be easily generalizable to other Spanish medical texts by re-training the network with additional data. However, the applicability of the de-identification methodology to other languages needs to be evaluated. We consider that our approach can be replicated in other Romance derived languages, following the training of a BiLSTM-CRF network with suitable data and the application of the randomization strategy. The easiest network to implement for deep learning non-specialized teams would be spaCy, although it is not the best performing. The proposed de-identification methodology still missed identifiers after training, thus a final check of the texts by an authorized person remains necessary. Nevertheless, we believe a combination of NER with the generation of synthetic data will make it virtually impossible to extract real identifying words from the text. Further efforts need to be done to assess and test this hypothesis.

## Data Availability

The data that support the findings of this study are available from BIMCV but restrictions apply to the availability of these data under a research use agreement. Data access can be requested at http://bimcv.cipf.es/bimcv-projects/dismed Supplementary information and code are available online in GitHub. • Project name: DiSMed - De-identifying Spanish medical texts • Project home page: https://github.com/BIMCV-CSUSP/DiSMed • Operating system(s): Platform independent • Programming language: Python • Other requirements: Python (version ≥3.5). DiSMed imports the following Python non-built-in libraries: pandas, numpy, codecs, spacy, tensorflow (version <2) • License: MIT

## Abbreviations

NLP: Natural language processing
NER: Named entity recognition
PHI: Protected health information
HIPAA: Health insurance portability and accountability act
NE: Named entity
BIMCV: Medical imaging databank of the Valencian Region
BiLSTM: Bidirectional long short-term memory
CRF: Conditional random fields
EMA: Exponential moving average
CNN: Convolutional neural network
